# Disseminating health evidence summaries to increase evidence use in health care

**DOI:** 10.11606/S1518-8787.2018052000418

**Published:** 2018-05-07

**Authors:** Maria Cristiane Barbosa Galvao, Fabio Carmona, Roland Grand, Pierre Pluye, Ivan Luiz Marques Ricarte

**Affiliations:** I Universidade de São Paulo. Faculdade de Medicina de Ribeirão Preto. Departamento de Medicina Social. Ribeirão Preto, SP, Brasil; II Universidade de São Paulo. Faculdade de Medicina de Ribeirão Preto. Departamento de Puericultura e Pediatria. Ribeirão Preto, SP, Brasil; III McGill University. Faculty of Medicine. Department of Family Medicine. Montreal, QC, Canada; IV Universidade Estadual de Campinas. Faculdade de Tecnologia. Limeira, SP, Brasil

**Keywords:** Evidence-Based Practice, Information Dissemination, Delivery of Health Care, Medical Informatics Applications, Public Health Informatics, Databases, Bibliographic, utilization

## Abstract

**OBJECTIVE:**

To verify whether an intervention based on disseminating health evidence summaries by e-mail to health professionals increases access to health evidence databases, and whether health professionals intend to apply the evidence received by e-mail in their clinical practice.

**METHODS:**

This quantitative study started with a survey to collect demographic data and patterns of access to health evidence databases. It was followed by a longitudinal intervention, over 48 weeks, that disseminated 143 health evidence summaries to 339 health professionals with higher education degree who work in the Brazilian Unified Health System. In the longitudinal intervention phase, health professionals voluntarily assessed the received health evidence summaries using the information assessment method. Finally, the study concluded with a survey to identify changes in accessing health evidence databases.

**RESULTS:**

Of the 339 Brazilian health professionals participating in this research, 90 (26.5%) answered the initial and final surveys. After 48 weeks, there was an increase in the use of health evidence databases; 186 (54.9%) participants submitted 7,942 assessments of health evidence summaries, which were relevant for patient care in 5,409 (68%) assessments.

**CONCLUSIONS:**

The dissemination of health evidence summaries by e-mail to health professionals in Brazil increases the reported use of evidence in clinical practice.

## INTRODUCTION

More than 20 years after reaching the mainstream scientific literature, evidence-based clinical practice is still far from fulfilling its potential^1^. Health professionals still point out several barriers to the effective use of up-to-date information in supporting clinical decisions, such as lack of resources, lack of time, inadequate skills, inadequate access, lack of knowledge, and financial barriers^2^. The adoption of evidence-based practice in resource-limited countries faces further barriers, such as the weak culture of knowledge seeking and sharing in the workplace^3^ and the need to educate health professionals to use evidence^4^ ^,^ ^5^.

To strengthen health systems and to improve the quality of healthcare, it is essential to provide information across all levels of the health system^6^. Aware of the need to provide up-to-date information to health professionals, in 2013 the Brazilian Ministry of Health established, in a partnership with the Pan-American Health Organization and the World Health Organization, a Web portal to promote evidence-based practice among Brazilian health professionals and academics. In the Brazilian Portal for Evidence-Based Health, more than 10 evidence databases are freely accessible by any Brazilian health professional or academic.

Nevertheless, merely having access to evidence databases may not be enough to promote evidence-based practice, since health professionals have little time to search and often ignore their need to search for more recent information^7^ ^,^ ^8^. Push technologies^9^, such as delivering clinical information by e-mail to health professionals, provide an alternative to overcome this limitation. Such approaches for disseminating information have been successfully used in developed countries^10^, but we know of no similar initiative in Brazil. This article presents the results of a study on the impact of pushing evidence by e-mail to Brazilian health professionals. The main objective was to verify whether an intervention based on pushing health evidence summaries to health professionals would increase the frequency of access to evidence databases. A secondary objective was to verify whether the health professional would apply the evidence that was pushed in their clinical practice.

## METHODS

Approved by an ethics committee, this quantitative study combined a before and after design with a longitudinal design. To begin, a survey collected demographic data and patterns of access to health evidence databases. This was followed by a longitudinal intervention that disseminated health evidence summaries for health professionals. In the longitudinal intervention, health professionals voluntarily assessed the received health evidence summaries. Finally, the study concluded with a survey to identify changes in accessing health evidence databases.

### Recruitment of Participants

The process of dissemination of the project and invitation of participants, supported by several health institutions, lasted 135 days. In September 2014, two meetings were held with the Health Department of the State of São Paulo and 19 regional health directors of the state. After these meetings, the Health Department of the State of São Paulo disseminated the project in different institutional channels of communication with health professionals. Regional councils of the health professions located in the State of São Paulo were contacted by e-mail, and then, the Regional Councils of Medicine, of Pharmacy, of Nutritionists, and of Physiotherapy and Occupational Therapy disseminated the project to their registered professionals. Additionally, health institutions such as the Virtual Health Library of the Pan American Health Organization, Associação dos Profissionais de Informação e Documentação em Ciências da Saúde do Estado do Rio de Janeiro, Agência de Notícias da Universidade de São Paulo and Fundação Oswaldo Cruz also disseminated the project. All participants signed the informed consent form.

### Inclusion Criteria and Participants Classification

This study was open to any graduate professional directly assisting patients in the Brazilian Unified Health System (SUS). The inclusion criteria, presented to potential participants in the invitations and in the informed consent form, were:

- To be a health professional;
- To hold a higher education degree;
- To work directly in-patient care in the SUS;
- To have an e-mail account;
- To have Internet access.

Participants agreed to answer two surveys (initial and final) and to receive three health evidence summaries per week for over 48 weeks and, if they so wished, they could assess the impact of each health evidence summary on their clinical practice by answering a short online form. Therefore, in this study, we classified participants into three groups:

- Group 1: participants that answered the initial survey and agreed to receive health evidence summaries for 48 weeks;
- Group 2: participants that answered the initial survey, agreed to receive health evidence summaries for 48 weeks, and assessed the impact of at least one health evidence summary; and
- Group 3: participants that answered the initial survey, agreed to receive health evidence summaries for 48 weeks, and answered the final survey.

Due to Brazilian law, all participants were volunteers and, therefore, could not receive any kind of incentive.

### Selection of Health Evidence for Intervention

The sources for selecting health evidence were the Brazilian Portal for Evidence-Based Health^a^, where Brazilian health professionals can freely access evidence databases (such as Access Medicine, Dynamed, Micromedex, Nursing Reference Center, and Rehabilitation Reference Center), and PubMed^b^. Members of the research team selected health evidence to be disseminated considering the epidemiology of diseases in Brazil, such as obesity, diabetes, cardiovascular disease, traffic accidents, dengue, and cesarean section.

Based on information in the available health evidence databases, members of the research team elaborated summaries, in Portuguese, presenting the text according to a template with:

- Title, expressed as a clinical question and also used as the subject for the e-mail messages;
- Summary of the evidence providing the answer to that clinical question;
- References for the sources from which the health evidence was extracted; and
- Identification of author, reviewer, and the person responsible for the health evidence summary dissemination.

Reviewing considered particularities of the SUS, such as free universal access to health care.

### Data Collection Instruments

This study used three distinct surveys. The first survey, applied at the beginning of the study, collected demographic data and data about habits of information access, posing the following central question: “What sources of information do you use to address questions about patient care?” Possible answers, non-exclusive, were: bibliographic databases, books, classifications (as ICD-10) and terminologies, conferences, health evidence databases, guidelines and calculators, another health professional, own knowledge, patient, patient health record, scientific articles, search engines, or specialized sites.

The second survey, used in the longitudinal design of the study, was the validated push version of the Information Assessment Method^11^ (IAM). The IAM questionnaire^c^ documents reflection on health information taken from health evidence databases. From the perspective of the health professional, the IAM questionnaire captures the value of information (how information is valuable from the users’ perspective). It documents the following four levels of outcomes of clinical information^12^ - its cognitive impact, its relevance for specific patients, and if relevant, any use and expected health benefit of this information - through four questions:

1. What is the impact of this information on you or your practice?
2. Is this information relevant for at least one of your patients?
3. Will you use this information for a specific patient?
4. For this patient, do you expect any health benefits as a result of applying this information?

In this study, research team members translated the IAM to Portuguese. This brief survey could be answered for each evidence summary sent to participants.

The third survey, applied at the end of the study, again addressed habits of information access, to document any change.

Study data were collected and managed using Research Electronic Data Capture (REDCap) electronic data capture tools^13^. Research Electronic Data Capture is a web-based application designed to support data capture for research studies, providing: 1) an interface for validated data entry; 2) audit trails for tracking data manipulation and export procedures; 3) automated export procedures for seamless data downloads to common statistical packages; and 4) procedures for importing data from external sources.

Descriptive statistics were used to analyze demographic data from the initial survey, to analyze the main sources of information for answering questions about patient care, and to analyze the impact of health evidence summaries on learning, on use for patient care and on expected benefits from the application of the information. Specifically, regarding this study's main objective, whether participants would look more for health evidence information after the study, absolute and relative change on reported access to health evidence databases in the initial and the final surveys were compared. Considering the second objective, to verify whether health evidence summaries have an impact on clinical practice, data from IAM assessments for each summary were analyzed, using Student's t-test, according to the following null hypothesis: Information contained in health evidence summaries sent by e-mail has no relevance for patients. Finally, responses to the IAM questionnaire were descriptively summarized according to types of cognitive impact, relevance for patients, use, and expected health benefits. All statistical analyses were performed using R^d^, version 3.3.1.

## RESULTS

Three hundred thirty-nine participants volunteered to answer the initial survey and to receive e-mail messages with health evidence summaries. According to their extent of participation in the study and the classification presented in the Methods section, the composition of the three groups of volunteers was:

- Group 1: 339 participants, 259 (76.4%) women and 80 (23.6%) men, aged from 18 to 63 (mean 38.0) years, and with up to 40 (mean 11.6) years of professional experience. They were from 93 distinct Brazilian cities in six different states.
- Group 2: 186 participants, 136 (73.1%) women and 50 (26.9%) men, aged from 21 to 63 (mean 38.2) years, with up to 40 (mean 11.8) years of professional experience. They were from 61 distinct cities from five Brazilian states.
- Group 3: 90 participants, 56 (62.2%) women and 34 (37.8%) men, aged from 21 to 62 (mean 39.0) years, with 1 to 37 (mean 12.5) years of professional experience. They were from 32 distinct Brazilian cities from three states, but 88 participants were from the state of São Paulo.

Table summarizes the distribution of participants by group and by profession.

**Table:** Distribution by profession of participants that answered the initial survey (Group 1), that assessed the impact of at least one health evidence summary (Group 2), and that answered the final survey (Group 3).

Profession	Group 1	Group 2	Group 3
	n (%)	n (%)	n (%)
Physician	74 (22)	39 (21)	27 (30)
Pharmacist	66 (19)	41 (22)	19 (21)
Nurse	62 (18)	35 (19)	13 (14)
Speech and language therapist	53 (16)	30 (16)	9 (10)
Physical therapist	17 (5)	11 (6)	4 (4)
Dentist	16 (5)	10 (5)	2 (2)
Psychologist	11 (3)	8 (4)	5 (6)
Social worker	8 (2)	4 (2)	2 (2)
Medical biologist	7 (2)	3 (2)	3 (3)
Nutritionist	7 (2)	1 (1)	2 (2)
Occupational therapist	6 (2)	3 (2)	2 (2)
Other profession	12 (4)	1 (1)	2 (2)

**Figure 1:**
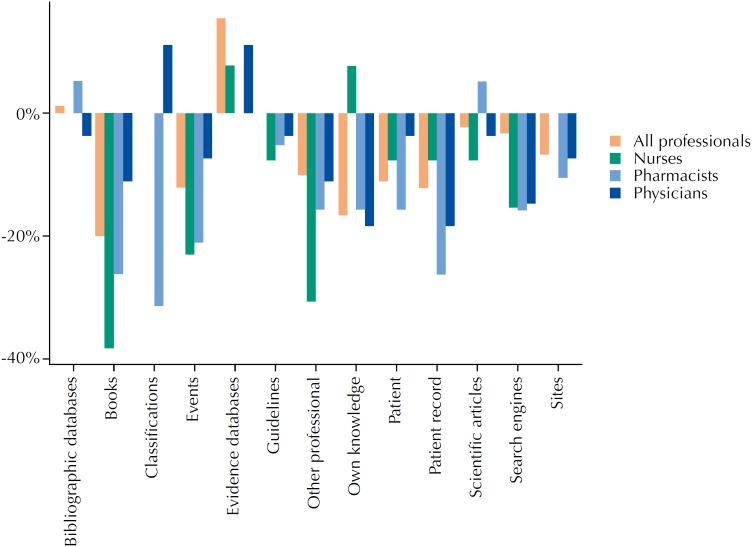
Relative change in the use of information sources by different professionals before and after receiving health evidence summaries.

Figure 1 presents how the 90 participants from Group 3 reported the change in the use of each information source, including health evidence databases. This data was collected from the following question: “What sources of information do you use to address questions about patient care?” The options were non-exclusive. Considering all professions, it can be observed that by the end of the study the increase of access to evidence databases was +16% (from n = 47 to n = 61), and the decrease of books was −20% (from n = 75 to n = 57) and own knowledge was −18% (from n = 58 to n = 43). In addition, the data indicate that changes in the use of information sources were not homogeneous among the different professions.

Demographic data were explored, and it was observed that age and years of professional experience do not correlate with change in the use of health evidence databases, with 95% confidence interval for correlation coefficient between −0.186 and 0.227 for age, and between −0.210 and 0.204 for experience.

**Figure 2:**
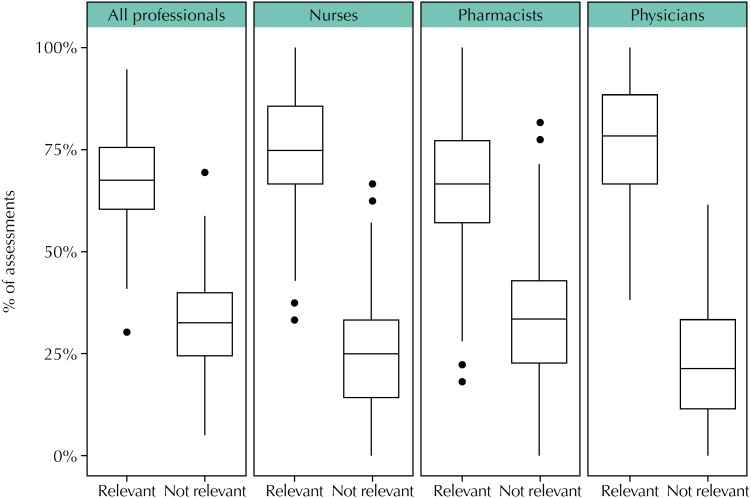
Perception by different professionals on the relevance of the information presented in health evidence summaries for patients.

Participants from Group 2 (n = 186) submitted 7,942 IAM questionnaires linked to 143 health evidence summaries. The relevance of the evidence for patient care was measured by the second question of IAM, “Is this information relevant for at least one of your patients”. To test the proposed null hypothesis (Information contained in health evidence summaries sent by e-mail has no relevance for patients), the distribution of assessments reported as “this information is relevant for at least one patient”, either totally or partially, were grouped and compared with assessments considering the information not relevant for patients. Considering all professions, from 7,942 assessments, 5,409 (68.1%) were relevant and 2,533 (31.9%) were not relevant. Nurses presented 1,194 assessments, with 931 (78%) relevant and 263 (22%) not relevant; pharmacists presented 1,623 assessments, with 1,100 (67.8%) relevant and 523 (32.2%) not relevant; and physicians presented 2006 assessments, with 1,555 (77.5%) relevant and 451 (22.5%) not relevant. These distributions are summarized in the box plots presented in Figure 2, which shows that the relevance of health summaries disseminated by e-mail was similar for the different professionals.

With these distributions of values, the null hypothesis was rejected (p < 0.01). Therefore, it is not true that delivering health evidence summaries by e-mail has no relevance for patients.

The IAM questionnaire captures other types of impact of information on the professional, and the following outcomes can be extracted from the assessments submitted by participants of Group 2.

**Figure 3:**
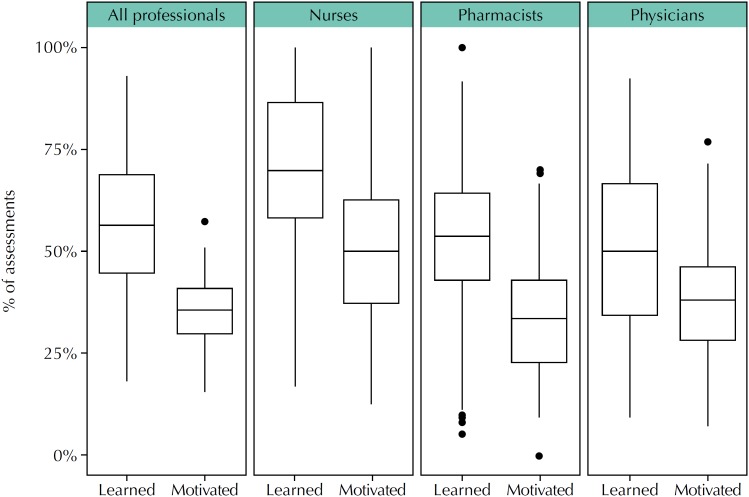
Perception by different professionals on the cognitive impact of information presented in health evidence summaries, considering the most frequent options, “I learned something new” (Learned) and “I am motivated to learn more” (Motivated).

The cognitive impact is captured by the first question of IAM, “What is the impact of this information on you or your practice?” Up to nine options can be chosen for this question, some capturing a positive impact (such as “I am motivated to learn more”) and some with negative impact (such as “I disagree with the content of this information”). From 11,789 responses received for this question in the 7,942 submitted assessments, the most frequent were “I learned something new”, with 4,621 (58.2%) responses, and “I am motivated to learn more”, with 2,788 (35.1%) responses. Nurses presented 1,194 assessments, being “I learned something new” 845 (70.8%) and “I am motivated to learn more” 564 (47.2%); pharmacists presented 1,623 assessments, such as “I learned” 859 (52.9%) and “I am motivated” 573 (35.3%); and physicians presented 2006 assessments, such as “I learned” 1,017 (50.7%) and “I am motivated” 748 (37.3%). These distributions are summarized in the box plots presented in Figure 3, which shows that health professionals, mainly nurses, have benefited from learning something new after receiving health evidence summaries by e-mail.

**Figure 4:**
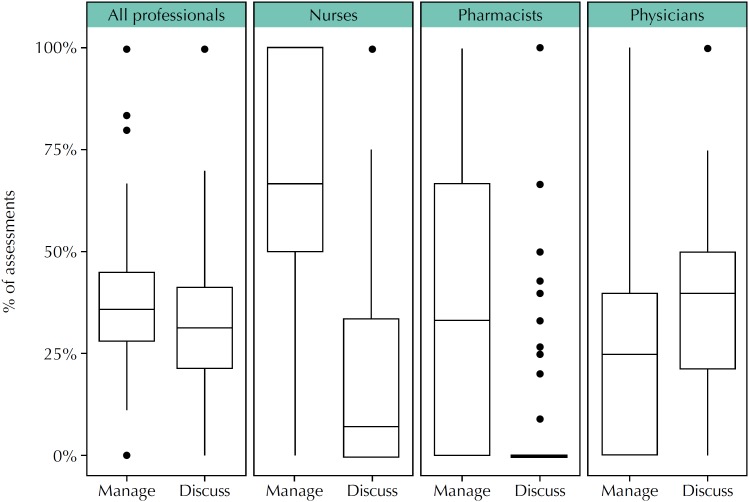
Perception by different professionals on the intended use of information presented in health evidence summaries, considering the most frequent options, “As a result of this information, I will manage this patient differently” (Manage) and “I will use this information in a discussion with this patient, or with other health professionals about this patient” (Discuss).

Participants who indicated that the information was relevant (either totally or partially) for at least one patient also answered the third IAM question, about the intended use of the information. From the 5,379 assessments that considered the information relevant for patients, 2,036 (37.9%) indicated that participants would use the information for a specific patient. The most frequent reported use of the information was “As a result of this information I will manage this patient differently”, with 721 (35.4%) responses, and “I will use this information in a discussion with this patient, or with other health professionals about this patient”, with 679 (33.3%) responses. As can be observed in the box plots in Figure 4, nurses (n = 458) tend to use more the information to manage patients differently (264, 57.6%) than to support discussions (121, 26.4%), whereas physicians (n = 655) use the information more for discussions (256, 39.1%) than to manage patients differently (171, 26.1%). Among pharmacists (n = 266), only 37 (13.9%) would use the information for discussions, and 101 (38.0%) to manage patients differently.

The final question of IAM is only answered by those that selected the option “Yes” to the third question (n = 2,036). The first part of this question asks if the professional expects any benefit from the use of this information, and 1,978 answers (97.2%) were affirmative. For these affirmative answers, the second part of the question asks about the expected type of benefit, with non-exclusive options. The most frequent answers were “This information will help to improve this patient's health status, functioning or resilience (i.e., ability to adapt to significant life stressors)”, with 1,084 (54.8%) responses, and “This information will help to prevent a disease or worsening of disease for this patient”, with 1,044 (52.8%) responses. The answer “help to improve” was most frequent among nurses (n = 450, 310 answers, 68.9%) and among pharmacists (n = 256, 146 answers, 57.0%), whereas among physicians (n = 642) the most frequent answer was “help to prevent” (345, 53.7%).

## DISCUSSION

The dissemination of health evidence summaries by e-mail (the so-called push approach) was associated with improved access to health evidence databases. These study results suggest that this approach is an effective way to disseminate evidence to health professionals and to promote evidence-based practice. Even in a heterogeneous group of health professionals, as in this study, participants considered that most of the evidence delivered by e-mail was relevant to their patients. More than a third of participants reported an intention to use the evidence in patient care and expected benefits for their patients.

It has been shown that this dissemination approach holds merit, and it can result in expected benefits for the patient. It can also be speculated that such intervention can lead to better resource use and to a decrease in the number of unnecessary tests and treatments. To our knowledge, this was the first attempt to push evidence to Brazilian health professionals. Similar results were found in a study developed in Canada, with the dissemination of e-mail evidence to more than 5,000 family medicine physicians. However, the participation of physicians in the study performed in the Canadian context provided credit for the annual registration of physicians in their College, which may have influenced the greater participation and adherence of Canadian professionals to this evidence-dissemination approach^10^.

Both human and technological resources were fundamental to implement this approach. Professionals from a variety of fields, such as Information Sciences, Information Technology, Medicine and the Health Sciences, worked together in an effective pipeline to select, summarize, review, disseminate, and collect feedback on clinical information needed at the local or regional level. From the technological point of view, required resources encompassed software to collect and analyze data, access to several health evidence databases, network and servers infrastructure, and, from participants, a valid e-mail account, and Internet access.

There are however limitations to this study. Despite all efforts to disseminate the project carried out in different communication channels, participants represented a small proportion of the total number of eligible health professionals. Furthermore, a selection bias was likely: professionals already committed to evidence-based practices may have been more prone to enroll in the study. Technological infrastructure of e-mail and Internet access in the health units, in São Paulo State, presents heterogeneous quality, which may have limited the participation of more health professionals in the study. Finally, a generalization of the study results to Brazilian states other than São Paulo cannot be made, since few participants were from other states. It should be noted that, following Brazilian regulations, participation in the study was voluntary and no type of incentive could be offered. To overcome these limitations, certainly, a more intensive partnership between researchers, universities, government agencies and professional councils may involve more health professionals to participate in this type of initiative.

## CONCLUSION

This research had the participation of 339 Brazilian health professionals, who received 143 health evidence summaries by e-mail over 48 weeks. The results indicate that the dissemination of health evidence summaries contributes to health professionals’ lifelong learning and is relevant to the care of their patients. Throughout the study, it was not observed that age or years of professional experience substantially interferes with the use of evidence in health. However, further investigation with this approach and in partnership with health institutions, professional councils, and universities is recommended so that there may be greater participation and adherence of health professionals to this type of study, as observed in the Canadian context.

## Footnotes

a Saúde Baseada em Evidências: base de dados. Brasília (DF): Ministério da Saúde; OPAS; c2016 [cited 2018 Jan 14]. Available from: shttp://psbe.ufrn.br/

b PubMed. Bethesda (MD): National Library of Medicine (US). 1946 [cited 2018 Jan 14]. Available from: http://www.ncbi.nlm.nih.gov/pubmed/

c Information Technology Primary Care Research Group. The Information Assessment Method. Montreal: McGill University, Department of Family Medicine; [c2018] [cited 2018 Jan 14]. Available from: http://www.mcgill.ca/iam

d R Foudation. The R Projet for Statistical Computing. Vienna; 2017 [cited 2018 Jan 14]. Available from: http://cran.r-project.org/
